# Assessment of dipyridamole stress echocardiography for risk stratification of diabetic patients

**DOI:** 10.1186/s12947-015-0030-7

**Published:** 2015-07-25

**Authors:** Liz Andréa Villela Baroncini, Rafael Borsoi, Maria Eugênia Bégué Vidal, Nathália Julim Valente, Juliana Veloso, Roberto Pecoits Filho

**Affiliations:** Center of Health and Biological Sciences, Pontificia Universidade Católica do Paraná, Rua Imaculada Conceição, 1155, Prado Velho, CEP: 80215-901 Curitiba, Paraná Brazil; Department of Internal Medicine, Medical School, Universidade Federal do Paraná, Rua XV de Novembro, 1299, Centro, CEP: 80060-000 Curitiba, Paraná Brazil; Medical School, Faculdade Evangélica, Rua Padre Anchieta, 2770, CEP: 80730-000 Curitiba, Paraná Brazil; Pontificia Universidade Católica do Paraná, ᅟ, ᅟ

**Keywords:** Stress echocardiography, Diabetes mellitus, Coronary artery disease

## Abstract

**Background:**

Despite advances in medical therapy, cardiovascular disease, mainly coronary artery disease (CAD), remains the leading cause of mortality among patients with diabetes mellitus (DM). The objective of the present study was to assess the effectiveness of dipyridamole stress echocardiography in identify diabetic patients at high risk for cardiovascular events.

**Methods:**

Dipyridamole stress echocardiography was administered to 483 diabetic patients (294 women; mean age 63.41 ± 11.28 years) between July 2006 and December 2012.

**Results:**

Follow-up data were available for 264 patients (163 women; mean age 64.3 ± 10.5 years): 250 with a negative stress echocardiography and 14 with a positive stress echo. During a mean follow-up time of 18 ± 14 months, a cardiovascular event occurred in 18 (6.8 %) patients, 12 (4.8 %) in patients with a negative stress echo (n = 250) during a mean follow-up period of 20 ± 16 months and 6 (42 %) in patients with positive stress echo (n = 14) during a mean follow-up of 13 ± 13 months. The positive and negative predictive values of stress echocardiography were 42 % and 96 % respectively. The accuracy value was 92 %. A Cox regression model showed that CAD (hazard ratio [HR] 5.4, 95 % confidence interval [CI] 1.9-15.4; *p* = 0.002) and positive stress echocardiography (HR 7.1, 95 % CI 2.5-20.5; *p* < 0.001) were significant predictors of cardiovascular events.

**Conclusions:**

For patients with diabetes, a negative dipyridamole stress echocardiogram predicts favorable outcome during the first year of follow-up. A new stress imaging test should be done after 12 months in diabetic patients.

## Background

Despite advances in medical therapy, cardiovascular disease, mainly coronary artery disease (CAD), remains the leading cause of mortality among patients with diabetes mellitus (DM). Indeed, DM has been classified as a coronary heart disease equivalent by both the American Heart Association and American College of Cardiology [1, 2]. In addition, diabetes is associated with a higher rate of progression of coronary lesions, coronary occlusion, plaque ulceration, thrombosis, and formation of new luminal narrowing, which suggests that the features of vascular disease are different in diabetic and nondiabetic patients [3]. The challenge is to identify these high-risk patients early in their disease using noninvasive imaging methods. In a study assessing 1123 patients with type 2 diabetes and no symptoms of CAD [4], the participants were randomly assigned to be, or not to be, screened with adenosine-stress radionuclide myocardial perfusion imaging (MPI). The authors concluded that the cardiac event rates were low and were not significantly reduced by MPI screening for myocardial ischemia over 4.8 years. However, the negative predictive value of stress echocardiography is thought to be lower in patients with DM. Diabetic patients with a normal stress echocardiogram have a higher risk for subsequent cardiovascular events than nondiabetic patients, especially in the second year after undergoing stress echocardiography [5, 6]. Unlike a normal or negative stress echocardiography, a positive echo stress test, or one which detects ischemia, enables the identification of patients at greater risk of cardiac events [7, 8]. Moreover, diabetic patients are more likely to have diffuse distal vascular disease. In these patients regional wall motion abnormalities of the left ventricle are harder to identify by stress echocardiography, because the reduced perfusion is global instead of regional [8–13]. Now there are new ultrasound imaging modalities that can identify early changes in the myocardium [13, 14]; however these modalities are not accessible to all patients and private clinics and routine screening of millions of asymptomatic diabetic patients would be prohibitively expensive. Most physicians use less expensive tests such as the electrocardiographic (EKG) exercise test and pharmacological stress echocardiography to stratify their patients’ risk. Therefore, the purpose of this study was to assess the effectiveness of dipyridamole stress echocardiography in identify diabetic patients at high risk for cardiovascular events.

## Methods

### Patients

We retrospectively assessed 483 consecutive diabetic patients (294 women), with a mean age of 63.41 ± 11.28 years, who underwent dipyridamole stress echocardiography between July 2006 and December 2012 in a private cardiological clinic. Each patient’s blood sample results and previous imaging exams were analyzed before the stress echo test was performed. Before the study, the ultrasonographist collected information on the patients’ demographic characteristics and risk factors, according to the blood sample results and the report by the private cardiologist. Patients were questioned about the presence of hypertension, DM, dyslipidemia, CAD, and current smoking habit. Hypertension was defined as a history of treated hypertension or the presence of systolic BP ≥ 140 mmHg or diastolic BP ≥ 90 mmHg as measured by the private cardiologist. Smoking history was coded as never or current smoker. Subjects were classified as having diabetes when treated for insulin-dependent or non-insulin-dependent diabetes or having elevated fasting glucose levels (≥126 mg/dL). Patients’ records included the use of lipid-lowering drugs or the presence of total cholesterol > 200 mg/dL, HDL-cholesterol < 40 mg/dL, LDL - cholesterol > 100 mg/dL or triglycerides > 150 mg/dL [15–17], as well as a history of myocardial infarction, angioplasty, or coronary artery bypass surgery was recorded. A positive CAD history was considered to be the presence of any of these previous conditions. No patient at the present study presented a history of stroke or transient ischemic attack or reported intermittent claudication suggesting peripheral arterial disease. The reported indications for the exam included referral from a physician, information from close relatives, or the complaints of the participating patient. The indication for a stress test and treatment were exclusively offered by the private cardiologist. Twenty-seven percent of the exams (n = 264) were performed for a routine clinical and imaging follow-up; i.e., diabetic patients without other identifiable associated risk factor and with no specific complaints. The second most frequent indication for testing was thoracic pain in 58 patients (22 %), followed by perioperative risk stratification in 41 patients (15 %), and assessment of known CAD (14 %). Other indications included changes in the resting EKG (7 %; atrial fibrillation, left bundle branch block, right bundle branch block, and ST segment abnormalities) and evaluation of dyspnea/fatigue (4 %). Seventy patients underwent treadmill EKG stress testing using Bruce protocol before pharmacologic stress echocardiography was performed. Twenty-one patients had inconclusive exercise EKG test results (they did not reach submaximal heart rate), and 49 patients had altered exercise EKG readings, suggestive of coronary ischemic disease. The patients’ baseline characteristics and the indications for pharmacological stress echocardiography are shown in Tables 1 and 2. Moreover, the tables include both the number of diabetic patients who underwent dipyridamole stress echocardiography (n = 483) and the actual number of patients from whom we obtained data at the follow-up period (n = 264). Written informed consent to undergo stress testing and to participate in the study was obtained from each patient.

**Table 1:** Patients’ baseline characteristics

Patients (N)	483	264
Sex (M/F)	189/294	101/163
Age (y ± SD)	63.41 ± 11.28	64.3 ± 10.5
History of hypertension (N/%)	407 (84 %)	223 (84.5 %)
History of dyslipidemia (N/%)	329 (68 %)	181 (68.6 %)
Coronary artery disease (N/%)	68 (14 %)	37 (14 %)
Current smoking (N/%)	51 (10 %)	29 (11 %)
Altered/insufficient exercise stress testing (N/%)	132 (27 %)	70 (26 %)
^1^Altered EKG (N/%)	30 (6 %)	19 (7 %)

^1^Altered EKG = atrial fibrillation, left bundle branch block, right bundle branch block, and ST segment abnormalities

**Table 2:** Indications for pharmacologic stress echo

Indications	483 -N(%)	264 - N(%)
Routine clinical and imaging follow-up	123 (25 %)	72 (27 %)
Thoracic pain	107 (22 %)	58 (22 %)
Perioperative risk stratification	76 (15 %)	41 (15 %)
Evaluation of known coronary artery disease	69 (14 %)	37 (14 %)
Altered resting EKG	30 (6 %)	19 (7 %)
Dyspnea/fatigue	18 (4 %)	12 (4 %)
Altered/insufficient exercise stress testing (N/%)	132 (27 %)	70 (26 %)

### Follow-up data

Follow-up data were obtained after a minimum of 6 months from telephone interviews with the patient or a close relative, or contact with the patient’s physician. The cardiovascular events recorded during the follow-up period were fatal and nonfatal myocardial infarction, unstable angina that required hospitalization, coronary revascularization procedures (surgery or angioplasty), and sudden death.

### Stress echocardiography protocol

An accelerated high-dose dipyridamole protocol was used for all patients. Dipyridamole was infused intravenously at a dose of 0.84 mg/kg body weight over 6 min. Aminophylline was routinely administered to patients with negative findings 10 min after the initiation of the test. Two-dimensional echocardiography and 12-lead electrocardiography (EKG) were used for continuous monitoring during the test and the recovery phase. Blood pressure measurements using a cuff were recorded every minute. Echocardiographic images were semiquantitatively assessed using a 17 segments, 4-point scale model of the left ventricle. Wall motion score index (WMSI) was derived by dividing the sum of individual segment scores by the number of interpretable segments. Ischemia was defined as stress-induced new and/or worsening of pre-existing wall motion abnormality, or biphasic response (i.e. low-dose improvement followed by high-dose deterioration). Inotropic reserve was defined as any improvement of WMSI during stress in the absence of inducible ischemia. Necrotic pattern was akinetic or diskinetic myocardium with no thickening during stress. An hypokinetic segment that did not worsen during ischemic challenge was considered a rest wall motion abnormality (WMA). A test was normal in case of no rest and stress WMA. A test was considered positive for ischemia when at least 2 adjacent segments of the same vascular territory showed an increment of WMSI (worsening or regional function) of at least 1 point at peak stress [18]. The following criteria were used for a premature interruption of the test: onset of obvious new wall motion abnormalities, severe chest pain, horizontal or downsloping ST-depression ≥ 2 mm, ST-segment elevation ≥ 1.5 mm, symptomatic hypotension and/or bradycardia, supra-ventricular or ventricular tachyarrythmias, and intolerable symptoms. Intravenous aminophylline (up to 240 mg) was immediately available to reverse the effects of dipyridamole. As recommended by Dal Porto et al. [19], each patient’s intravenous cannula remained in situ for 1 h after the stress test in the event of a late event occurring after the procedure. All the tests were performed by the same echocardiographer, who had more than 15 years of experience.

### Statistical analyses

Quantitative variables were expressed as mean, median, and standard deviation, and qualitative variables as frequencies and percentages. Positive and negative predictive values and accuracy were calculated according to standard formulas. Kaplan-Meier survival estimations were performed for age, hypertension, smoking, CAD, and results of stress testing. The log-rank test was used to compare survival curves. An adjusted Cox regression model was used for multivariate analysis of variables, considering stress echo result and the presence of CAD as explanatory variables, with *p*-values <0.05. *P*-values <0.05 indicated statistical significance. Data were analyzed using the software program SPSS v. 20.0.

## Results

### Stress echocardiography result

Two-hundred fifty patients had presented negative dipyridamole stress echocardiography results. Of the 14 patients (5 %, n = 264) with positive stress echocardiography, 7 had undergone the procedure as a routine clinical and imaging follow-up, 5 for chest pain, 1 for an inconclusive exercise EKG test, and 1 for heart failure. Of the 70 patients who had undergone an exercise EKG test before pharmacological stress echocardiography, only 1 presented positive dipyridamole stress test and 3 patients developed a cardiovascular event during the follow-up period.

### Follow-up data

**Fig. 1:**
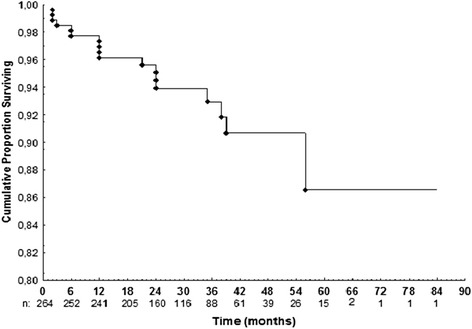
Cumulative proportion survival curve for cardiovascular events

**Fig. 2:**
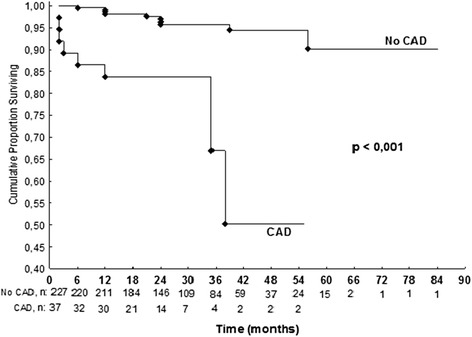
Cumulative proportion survival curve for the presence of coronary artery disease

**Fig. 3:**
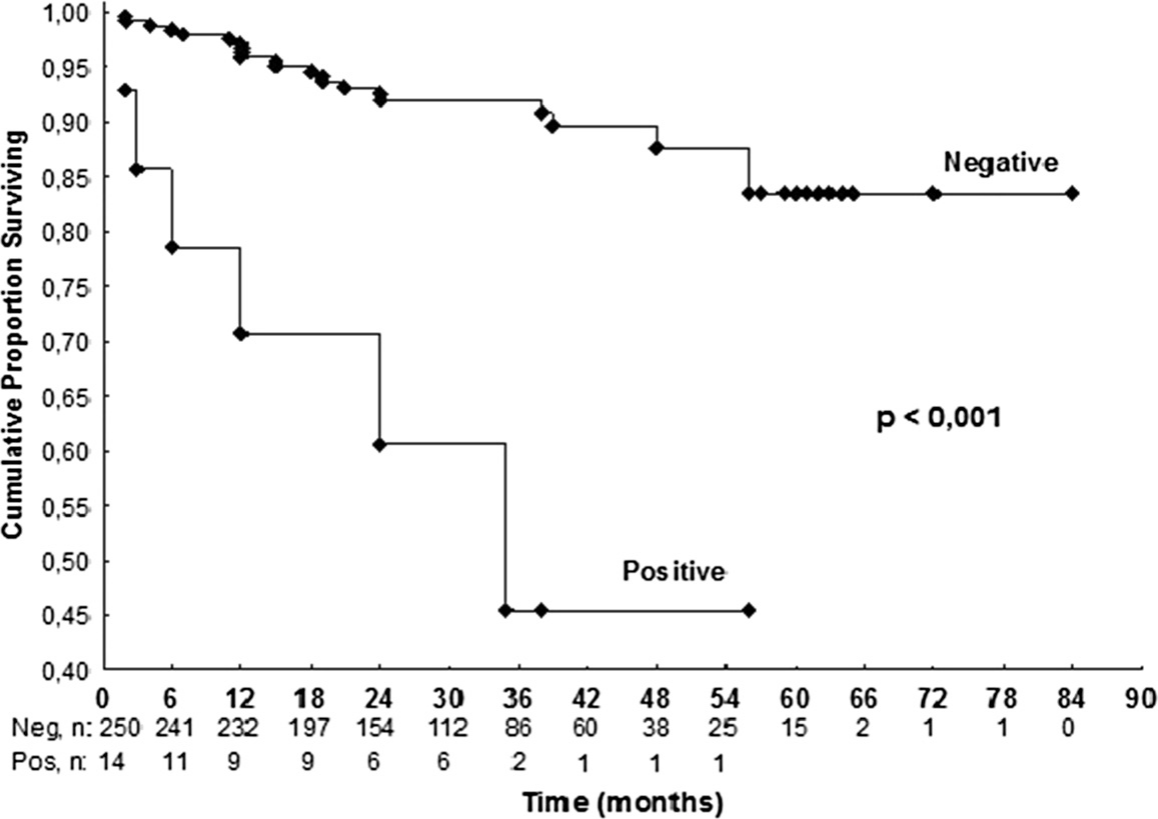
Cumulative proportion survival curve for positive stress echocardiography

We were unable to establish contact with 219 patients. We attribute that to changes in telephone network rules. Follow-up data were available for 264 patients (163 women; mean age 64.3 ± 10.5 years, mean duration of follow-up 32.7 ± 15 months, median duration 30 months, minimum 6 months). There were 18 cardiovascular events including cardiovascular deaths during the follow-up period. Cardiovascular deaths included the following: 1 sudden death 24 months after a negative stress test, 1 after surgical myocardial revascularization 2 months after a negative stress test, 1 due to acute myocardial infarction 12 months after a negative stress test, and 1 due to acute myocardial infarction 24 months after a positive stress test. There were 14 other cardiovascular events (nonfatal myocardial infarction, unstable angina that required hospitalization, and coronary revascularization procedures) in 14 patients during follow-up: 9 after a negative stress echo test and 5 after a positive stress echo test. The positive predictive value of stress echocardiography was 42 % and the negative predictive value was 96 %. The accuracy was 92 %. We did not calculate the sensitivity and specificity of the test in this study, because most patients did not undergo coronary angiography, even after a positive stress test. The referring physician made the decision whether or not to perform coronary angiography. Of 250 patients with a negative stress test, there were 12 (4.8 %) in whom a cardiovascular event occurred during a mean follow-up period of 20 ± 16 months. Most events occurred at least 1 year after the test. In those 14 patients (5 %, n = 264) with positive stress echocardiography there were 6 events (42 %, n = 14) during a mean follow-up of 13 ± 13 months. In those patients with known CAD there were 10 % of cardiovascular events during the follow-up period compared to 5 % of cardiovascular events in the patients without CAD. Univariate analysis for all cardiovascular events showed that there was no significant difference for gender, age (cut-off, 65 years), hypertension, dyslipidemia, or smoking. A Cox regression model showed that CAD (hazard ratio [HR] 5.4, 95 % confidence interval [CI] 1.9-15.4; *p* = 0.002) and positive stress echocardiography (HR 7.1, 95 % CI 2.5-20.5; *p* < 0.001) were significant predictors of cardiovascular events (Table 3; Figs. 1, 2, and 3).

**Table 3:** Risk of cardiovascular events according to Cox regression model for multivariate analysis

Variable	p*	p**	HR (IC 95 %)
Age ≥ 65	0.998		
Sex	0.636		
Current smoking	0.969		
Dyslipidemia	0.171		
Hypertension	0.147		
Coronary artery disease	<0.001	0.002	5.4 (1.9-15.4)
Positive stress echo	<0.001	<0.001	7.1 (2.5-20.5)

*Log-rank test, *p* < 0.05

**Cox Regression Model and Wald test, *p* < 0.05

*HR* hazard rate

## Discussion

The utility of stress testing modalities for asymptomatic diabetic patients remains an area of active interest and study. Diabetic patients have significantly higher rates of silent ischemia than the general population, and that could in part explain the more advanced CAD seen on initial presentation and the worse outcomes in these patients [20]. The absence of myocardial ischemia on noninvasive tests of patients with DM does not necessarily identify a lower-risk cohort. In the present study most of patients (250) had a negative stress echo and presented 4.8 % of cardiovascular event during the follow-up period. This may be related, at least in part, to the observation that diffuse coronary dysfunction in diabetic patients precedes overt atherosclerosis and regional wall motion abnormalities of the left ventricle are harder to identify by stress echocardiography, because the reduced perfusion is global instead of regional [1, 8–13]. Thus, the current guidelines recommend that all diabetic patients should be treated as if they have CAD with regard to blood pressure management, lipid-level goals, and other secondary preventive measures. Clinicians should make efforts to stratify the long-term risk of CAD-associated morbidity and mortality in diabetic patients in order to identify those patients who need more aggressive treatment strategies. The guidelines of the American College of Cardiology/American Heart Association and the American Diabetes Association recommend screening for CAD in diabetic patients with an abnormal resting EKG indicating myocardial infarction, with carotid or peripheral arterial disease, symptoms suggesting CAD, or 2 or more cardiovascular risk factors irrespective of the presence of CAD symptoms [21–22]. However, as seen in this study, these guidelines do not seem to satisfy physicians regarding the risk stratification of diabetic patients. Our results showed that 25 % of patients underwent pharmacological stress echocardiography with the only indication being that they had DM and stress echo is a safe procedure [23]. The annual rate of hard events occurring in diabetic patients with a normal stress echocardiogram ranges from 1.6 % to 6 %, whereas the corresponding rate in nondiabetic patients ranges from 0.6 % to 2.7 %. In addition, in patients with DM the event rate increases sharply in the second year after the procedure [24]. In our study, 4.8 % of patients had an event during a mean follow-up period of 18 ± 14 months in patients with a negative stress test, most occurring 1 year after the test. By contrast, among those patients with a positive stress test, there were about 4-fold more events during a mean follow-up of 13 ± 13 months. These findings are similar to other studies [12, 25–26]. Cortigiani et al. [5] compared the prognostic value of pharmacologic stress echocardiography in chest pain patients with and without DM, (mean age 60 ± 10 years) and positive exercise electrocardiography. During a median follow-up of 26 months, the results of stress echocardiography added prognostic value to the positive exercise electrocardiography results. Both diabetic and nondiabetic patients with nonischemic stress echocardiography results had lower annual rates of major events compared with the overall population of diabetic and nondiabetic patients with positive electrocardiography results. However, in our study, most patients did not undergo exercise stress testing before pharmacological stress echocardiography. Patients referred for pharmacological stress echocardiography have been found to have a higher risk for cardiovascular events than those referred for exercise testing, which likely reflects more severe underlying cardiovascular disease and comorbidities [12]. When our patients were asked why they did not undergo an EKG exercise stress test before the pharmacological stress test, most answered that their private physician asked directly for a pharmacological test regardless of whether they could undergo an exercise test. Of the 70 patients who underwent exercise EKG testing before pharmacological echocardiography only 1 had a positive dipyridamole stress test and we found only three cardiovascular events during the follow-up period in these patients. It appears that physicians simply do not believe that an exercise test is adequate for stratifying their diabetic patients. In our study, the time to occurrence of cardiovascular events was significant shorter in patients with a positive echocardiography stress test compared to patients with a negative stress test (13.7 ± 13.2 versus 20.7 ± 16.6 months, respectively). However, most cardiovascular events, regardless of the results of stress testing, occurred around 18 months after the procedure, which suggests that patients with DM should undergo another imaging stress test 12 months after their first test. In a study assessing long-term outcomes of patients with diabetes (N = 230) and limited exercise capability, dobutamine stress echocardiography provided prognostic value for about 7 years after the initial test [27]. However, in both that study and our study, the lack of data on the duration of diabetes is an important shortcoming. In addition, the socioeconomic background of the 2 study cohorts may be different, and that factor can affect the prognosis and evaluation of the disease.

### Study limitations

The main limitations of this study include: (1) loss of > 50 % of patients in the follow-up period; (2) lack of information about each patients’ diabetes time course or renal function; (3) unknown exact number of subjects under anti-ischemic medical therapy; and (4) lack of data on patients’ blood pressure control and medication. These limitations could significantly influence our analysis and discussion and may have impacted the results of this study [28–34].

## Conclusions

A negative dipyridamole echocardiography test in diabetic patients predicts favorable outcome for the first year of follow-up. A new stress imaging test should be done after 12 months in diabetic patients.
